# Field evaluation of Mosq-ovitrap, Ovitrap and a CO_2_-light trap for *Aedes albopictus* sampling in Shanghai, China

**DOI:** 10.7717/peerj.8031

**Published:** 2019-11-27

**Authors:** Qiang Gao, Hui Cao, Jian Fan, Zhendong Zhang, Shuqing Jin, Fei Su, Peien Leng, Chenglong Xiong

**Affiliations:** 1 Department of Vector Control, Shanghai Huangpu Center for Disease Control & Prevention, Shanghai, China; 2 Department of Vector Control, Shanghai Municipal Center for Disease Control & Prevention, Shanghai, China; 3 Department of Epidemiology, School of Public Health, Fudan University, Shanghai, China

**Keywords:** Ovitrap, CO_2_-light trap, Mosq-ovitrap, *Aedes albopictus*, downtown Shanghai

## Abstract

**Background:**

The Mosq-ovitrap (MOT) is currently used for routine surveillance of container-breeding *Aedes* in China. However, the effectiveness of monitoring* Aedes albopictus* using the MOT and other mosquito monitoring methods, such as the Ovitrap (OT) and the CO_2_-light trap (CLT), have not been extensively compared. Moreover, little is known about the spatial-temporal correlations of eggs with adult *Ae. albopictus* abundance among these three types of traps.

**Methods:**

Comparative field evaluation of MOT, OT and CLT for *Ae. albopictus* monitoring was conducted simultaneously at two city parks and three residential neighborhoods in downtown Shanghai for 8 months from April 21 to December 21, 2017.

**Results:**

Significantly more *Ae. albopictus* eggs were collected from both MOTs and OTs when traps remained in the field for 10 d or 7 d compared with 3 d (MOT: 50.16, 34.15 vs. 12.38 per trap, *P* < 0.001; OT: 3.98, 2.92 vs. 0.63 per trap, *P* < 0.001). Egg collections of MOTs were significantly greater than OTs for all three exposure durations (Percent positive: *X*^2^ = 72.251, 52.420 and 51.429, *P* value all < 0.001; egg collections: *t* = 8.068, 8.517 and 10.021, *P* value all <0.001). Significant temporal correlations were observed between yields of MOT and CLT in all sampling locations and 3 different MOT exposure durations (correlation coefficient r ranged from 0.439 to 0.850, *P* values all < 0.05). However, great variation was found in the spatial distributions of *Ae. albopictus* density between MOT and CLT. MOT considerably underestimated *Ae. albopictus* abundances in areas with high *Ae. albopictus* density (>25.56 per day ⋅ trap by CLT).

**Conclusion:**

The MOT was more efficient than the OT in percent positive scores and egg collections of *Ae. albopictus*. The minimum length of time that MOTs are deployed in the field should not be less than 7 d, as *Ae. albopictus* collections during this period were much greater than for 3 d of monitoring. MOT considerably underestimated *Ae. albopictus* abundance in areas with high* Aedes albopictus* density compared to CLT. In areas with moderate *Aedes albopictus* densities, MOT results were significantly correlated with CLT catches.

## Introduction

*Aedes albopictus* (Skuse, 1894) (Diptera: Culicidae) is the predominant and the most important arbovirus vector species in the Shanghai region of eastern China (Gao et al., 2014; Zhou et al., 2015). This species is now a top priority of vector control efforts in Shanghai and surrounding areas, especially after the first autochthonous dengue case was recently reported (SHWSJKW, 2017). Vector monitoring is used to estimate *Ae. albopictus* density and biting rates. However, adult mosquito monitoring approaches are often labor-intensive, unethical (i.e., human landing catches), expensive and difficult to implement on a large scale (i.e., BG sentinel traps and CO_2_-light traps) (Manica et al., 2017). Artificial traps for *Aedes (Stegomyia)* egg collection (i.e., Ovitrap, Mosq-ovitrap) are relatively easy and inexpensive to construct. They are also easier to implement in the field because they are portable and do not require electricity or CO_2_. Egg traps can detect the presence of gravid *Aedes* females, even at low population densities. This makes them good alternatives for *Aedes* monitoring (Silver, 2008). If statistical correlations between egg-collection data and adult population density can be demonstrated, then the workload and cost for adult *Aedes* monitoring could be reduced.

The Ovitrap (OT) was initially developed during the first 3 years of the USA *Ae. aegypti* Eradication Program. It was used to detect the presence of *Ae. aegypti* (Fay & Eliason, 1966; Fay & Perry, 1965; Jakob & Bevier, 1969). The OT is now a common *Aedes* monitoring approach in many regions, including North and South America, Europe and Asia, due to its high sensitivity and low implementation costs (ECDC, 2012; Mogi et al., 1988; Reiter, Amador & Colon, 1991). Mattia (Manica et al., 2017) demonstrated the possibility of predicting the biting rate of *Ae. albopictus* based on OT egg-collection data. However, the reliability of OT data for estimating adult population abundance has been debated (Focks, 2003). The Mosq-ovitrap (MOT), based on the OT, was developed by Lin et al. (2005). It is a safe, efficient and economical method for conducting *Aedes* surveillance. The trap design makes it difficult for captured gravid *Aedes* females to escape, so MOT can quantitatively measure both adult females and their egg production.

The MOT was developed, and is mainly used, in China. Its field sampling efficiency, in comparison to other traps, is poorly known. Lin et al. (2006b) demonstrated positive correlations between MOT and OT for some macro indices, such as the positive index (percentage of traps containing adult mosquitoes or eggs), but detailed indices, including egg counts collected by the two traps, were not compared. The MOT has been widely used for routine *Ae. albopictus* monitoring in China (Li et al., 2016), and the mosq-oviposition positive index (MOI) lower than 5 has been used as the threshold for *Ae. albopictus* population density being less than the level requiring control treatment. The sampling efficiency of the MOT has not been extensively compared with other adult mosquito monitoring methods, such as human landing catch or CO_2_-light trap (CLT). (Li et al., 2016) compared MOT with the BG-trap and the CDC light trap, but, due to the small number of captures, the MOT data were not analyzed. Therefore, little is known about the spatial–temporal correlations of eggs or adult mosquito catches between MOT and adult mosquito traps. The surveillance duration of MOT in China is typically 3 d compared to the widely accepted 7 d interval for field surveillance using CDC ovitraps (Reiter, Amador & Colon, 1991). It is not known if the field surveillance time for MOT should be extended. It is also unknown if the low mosquito capture efficiency of MOT in the Li et al. (2016) study was caused by the 3 d exposure duration.

To address these questions, we conducted a comparative field evaluation of MOT, OT and CLT for *Ae. albopictus* monitoring in urban areas of Shanghai. We determined the best sampling duration for MOT to maximize *Ae. albopictus* catches and then conducted correlation analysis and efficiency evaluations between MOT and OT. For adult mosquito density, spatial–temporal correlations of eggs or adult mosquito catches between MOT and adult mosquito trap CLT were made. The advantages and disadvantages of MOTs used as surveillance tools for *Ae. albopictus* population level were then described.

## Materials and Methods

### Study area

**Figure 1:**
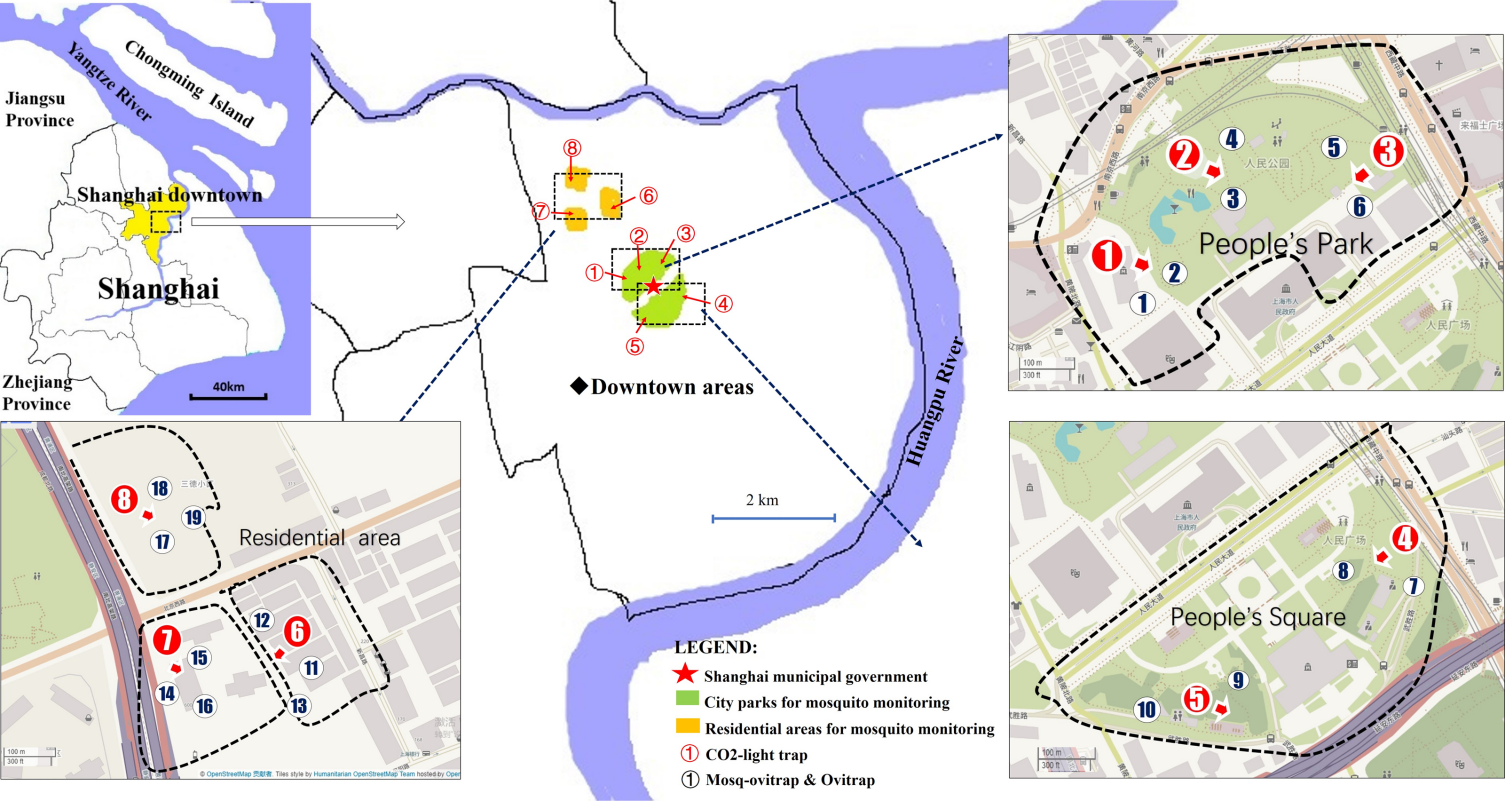
Trap locations for mosquito monitoring comparisons of MOT, OT and CLT. Map data 2019 OpenStreetMap.

The study was conducted in 5 downtown areas of Shanghai, China (31°13′N, 121°27′E, el 3.5 m). The areas consisted of 2 city parks and 3 residential neighborhoods (Fig. 1). A total of 8 field sites were used for comparisons among the CLT, MOT and OT trap. Detailed geographical coordinates of the 8 sites were described in a previous report (Gao et al., 2016). We placed one CLT (total = 8 CLT) and 2 or 3 pairs (total = 19 pairs) of MOTs and OTs in each site for comparison. To reduce direct competition among the traps, all traps within one site were separated by an average distance of 20 m.

### Traps tested

**Figure 2:**
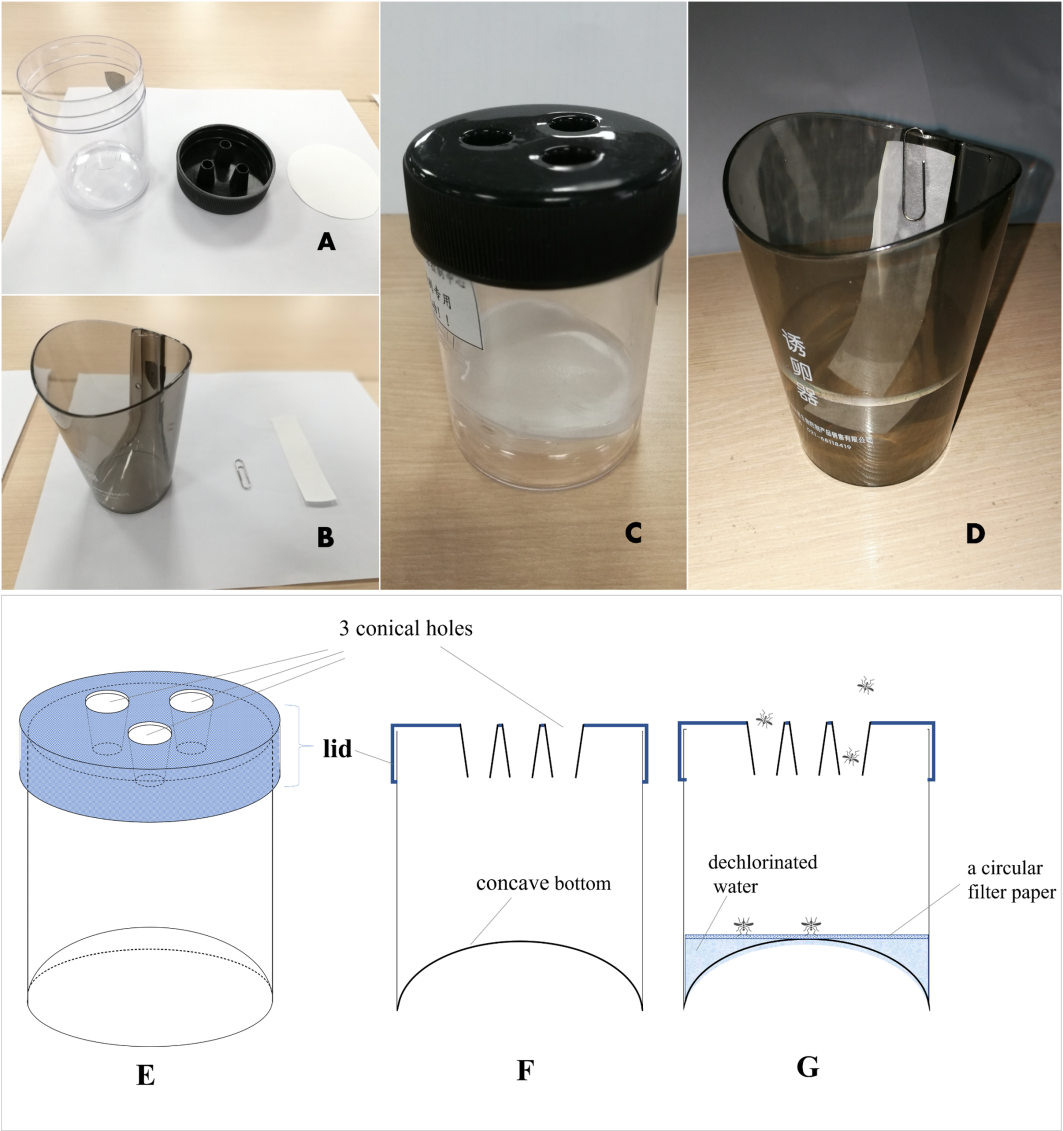
Mosq-ovitrap (A and C) and Ovitrap (B and D) used in this study; (E): a three-dimensional diagram of MOT; (F): a cross-sectional diagram of MOT; (G): a cross-sectional diagram of MOT with water and filter paper.

The MOT (Tianpai, Kaiqi Co. Ltd, Shanghai, China) used was described by Lin (Lin et al., 2005). It consists of a transparent cylindrical plastic jar (10 cm high ×7 cm diam.) with a concave bottom (about two cm inward) and a black top cover with 3 conical holes (one cm diam.) (Fig. 2). A white circular filter paper (7.5 cm in diameter) purchased from Aoke Co. Ltd (Taizhou, China) is placed inside the bottom of the jar as an oviposition substrate, and 20 ml of dechlorinated tap water is added to the jar to keep the filter moist but not submerged. The conical holes in the cover are designed for easy entry but difficult exit for the mosquitoes. Consistent with the observations of Lin (Lin et al., 2005), we also noted that captured gravid mosquitoes typically do not escape (escape rate was <10%, 15/162 = 9.26%) (Q Gao, 2016, unpublished data), and they will lay eggs on the paper substrate inside the trap. Therefore, this trap can provide a quantitative measure of the number of adult female mosquitoes and egg production. MOTs were placed on the ground in relatively secluded locations near vegetation and protected from rain.

The OT used in this study was purchased from Tianpai, Kaiqi Co. Ltd. (Shanghai, China). It consists of a black tinted plastic jar with straight, slightly tapered, sides (Fig. 2). The OT is approximately 10 cm high with an opening diameter of seven cm. A plastic paddle is affixed to inside wall of the jar, with an overflow hole three cm from the top. About 118 ml of dechlorinated water was added to the jar, and a white filter paper strip (12 × two cm) was used as an oviposition substrate.

**Figure 3:**
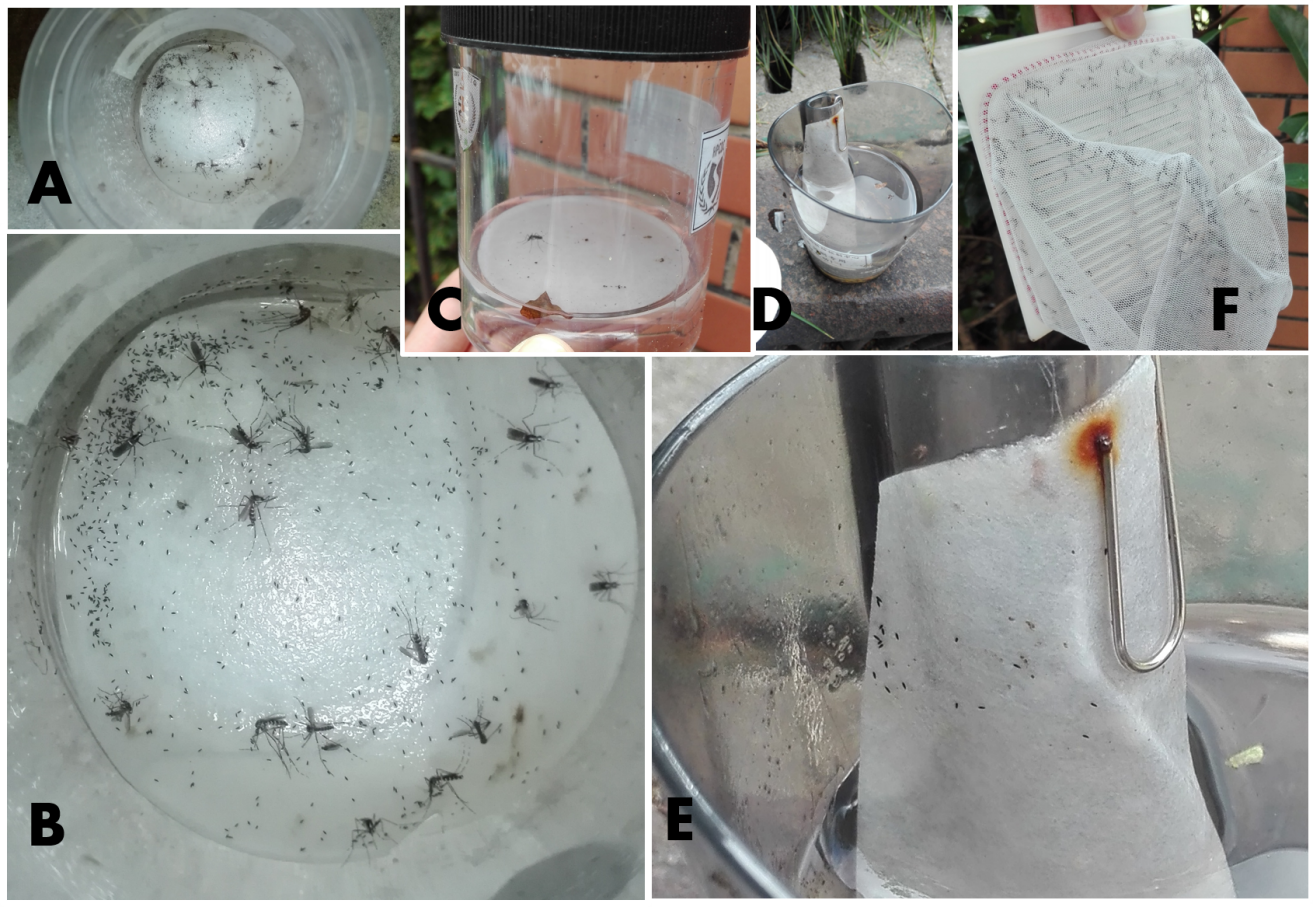
Field mosquito collection in three traps. (A, B, C) adult mosquitoes and eggs trapped by Mosq-ovitraps; (D, E) eggs trapped by ovitraps; (F) adult mosquitoes trapped by CO_2_-light traps.

The CLT used in this study was structurally similar to the Center for Disease Control and Prevention light-traps (Bat King Co. Ltd, Shanghai, China). The trap consists of an AC/battery-powered fan, a trap bag and an ultraviolet lamp (Fig. 3). We also used a compressed CO_2_ gas cylinder (5 kg) and a trap bait, produced by Bat King, that simulated human body scent. The CO_2_ gas cylinder and trap bait were replaced every 60 d.

### Mosquito sampling

Mosquito sampling was conducted during 8 months from April 21 to December 21, 2017. MOTs and OTs were left in the field for 10 d. The number of adult mosquitoes trapped and the number of eggs produced and trap conditions (i.e., tipped, dried, missing, flooded or broken) were recorded after 3, 7 and 10 d, respectively, for each sample interval. Collections were conducted between 0900 and 1200 h, which is the daily time of lowest mosquito activity (Haddow & Gillett, 1957; Reiter, Amador & Colon, 1991). Positive MOT or OT was defined as those containing at least one adult, egg or larva in the trap. CLT sampling of adult mosquitoes was conducted for 24 h every 10 d during the 8 months. The trapped mosquitoes were collected and stored at −80 °C.

### Measure of field mosquito collection yields

MOTs collect both adult mosquitoes and eggs. Since the collection yields were checked 3 times (after 3, 7 and 10 d), the number of mosquitoes and eggs trapped by MOTs could be field counted when mosquito abundance was relatively low. However, it was difficult to conduct accurate counts when there were large numbers of eggs. In this case, high definition photos were taken of the eggs in the traps, and the egg number was determined later by magnifying the photos. This method was also applied to egg collection measurement for the OTs. Mosquitoes collected by CLT were taken to the laboratory for identification and processing.

We used three measurements of mosquito trap sampling: percent positive, egg collection and mosquito density. “Percent positive” is defined as the percentage of positive MOT or OT in the total traps used for sampling (a positive trap has at least one egg, adult or larva). “Egg collection” refers to the number of eggs collected by traps, and “Mosquito density” refers to the density of trapped adults.

### Mosquito processing

Adult mosquitoes trapped by CLTs or MOTs were killed by freezing and then counted and identified using taxonomic keys (Becker et al., 2003). Eggs or eclosed larvae collected by MOTs and OTs after 10 d were taken back to the laboratory. For species identification, we randomly selected 20% of the trapped eggs or larvae and then hatched and reared these until adult emergence. Adults were identified and the results were extrapolated to the rest of the unhatched eggs.

### Statistics

Data were analyzed using SPSS ver. 11.5 (SPSS, Inc., Chicago, IL, USA) statistical software package. There were different measurements for mosquito collection in this study, including percent positive (%), egg collection (per trap) and mosquito density (per trap). The variance of percent positive (%) among three exposure durations was compared by using the Kurskal-Wallis H test, and variances in the different trap types were compared by Pearson chi-square test. For quantitative data of “egg collection” or “mosquito density,” independent *t* test or one-way ANOVA was used for analysis and Bonferroni test for pairwise comparisons. Correlation analysis was used for trend comparison between the different trap types. *P* < 0.05 represented a significant difference.

## Results

### Mosquito population structure based on captures of the three traps

A total of 21,919 and 1,740 eggs were collected by the MOT and OT, respectively. A randomized 20% of these eggs were reared to adults, and all were *Ae. albopictus*. A total of 370 adult *Ae. albopictus* (♀: ♂ = 355:15) and 3 adult female *Culex pipiens* complex were collected by the MOT; the *Cx. pipiens* were only trapped in city park II in mid-November. Four species were collected by CLTs, including 4,858 *Ae. albopictus* (♀: ♂ = 3,007:1,851), 475 *Cx. pipiens* complex (♀: ♂ = 314:161), 338 *Culex tritaeniorhynchus* ( ♀: ♂ = 335:3), and 9 ♀ *Anopheles sinesis*. It is not within the scope of this paper to present results for other mosquito species from the 3 traps; and so the results are limited to *Ae. albopictus.*

### Comparison between MOT and OT

For the two measurements of percent positive and egg collections (Table S1), MOT yields were significantly greater than OTs for all of the three exposure durations (percent positive: X ^2^ = 72.251, 52.420 and 51.429, *P* values all <0.001; egg collection: *t* = 8.068, 8.517 and 10.021, *P* values all <0.001) (Tables 1 and 2).

**Figure 4:**
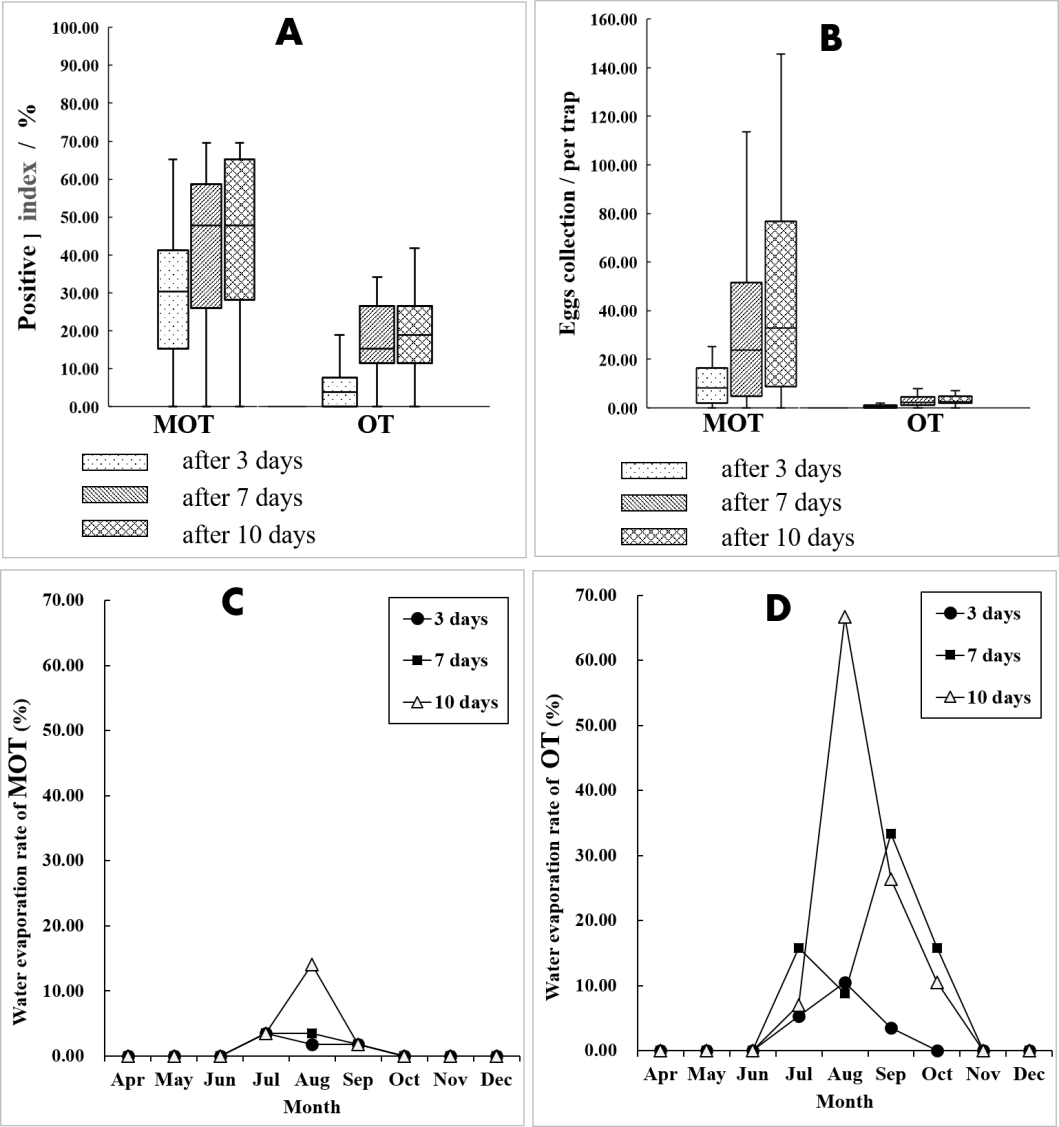
Comparation of mosquito collections between MOT and OT. (A and B) Comparison of mosquito collection indices between MOT and OT with different exposure durations. (C and D) Seasonal dynamics of water evaporation rate (%) of MOT and OT with different exposure durations.

For both MOT and OT, the percent positive and egg collections both significantly increased with increased exposure duration (Kruskal-Wallis H test for percent positive: *X*^2^ = 7.050, *P* = 0.029 of MOT, *X*^2^ = 17.678, *P* < 0.001 of OT; one-way ANOVA for egg collection: *F* = 4.854, *P* = 0.012 of MOT, *F* = 7.632, *P* = 0.001 of OT). At 7 d, the percent positive was significantly greater than the percent at 3 d (MOT: 42.33 vs. 28.15%, X^2^ = 19.272, *P* < 0.001; OT: 19.68 vs. 6.41%, X^2^ = 33.935, *P* < 0.001) (Fig. 4A). This was also true for the yield of egg collections (MOT: 34.15 vs. 12.38 per trap, *P* < 0.001; OT: 2.92 vs 0.63 per trap, *P* < 0.001) (Fig. 4B). A lesser increase of percent positive was observed at 10 d exposure compared with 7 d for both MOT and OT (MOT: 44.85 vs. 42.33%, X ^2^ = 0.563, *P* = 0.453; OT: 21.97 vs. 19.68%, X ^2^ = 0.694, *P* = 0.405). Egg collections were significantly greater at 10 d compared with 7 d for MOT (*P* = 0.011), but not significant for OT (*P* = 0.229) (Table 2, Figs. 4A and 4B).

**Table 1:** Comparisons of percent positive for traps with different types and different exposure durations. (a) MOT of 3 d; (b) MOT of 7 d; (c) MOT of 10 d; (d) OT of 3 d; (e) OT of 7 d; (f) OT of 10 d.

	Positive index I	Positive index II	X^2^ value (chi-square)	*P* value
MOT vs. OT with the same exposure durations	**a**: 28.15%	**d**: 6.41%	72.251	<0.001
	**b**: 42.33%	**e**: 19.68%	52.420	<0.001
	**c**: 44.85%	**f**: 21.97%	51.429	<0.001
MOT: Different exposure durations	**a**: 28.15%	**b**: 42.33%	19.272	<0.001
	**a**: 28.15%	**c**: 44.85%	26.307	<0.001
	**b**: 42.33%	**c**: 44.85%	0.563	**0.453**
OT: Different exposure durations	**d**: 6.41%	**e**: 19.68%	33.935	<0.001
	**d**: 6.41%	**f**: 21.97%	43.456	<0.001
	**e**: 19.68%	**f:** 21.97%	0.694	**0.405**

**Table 2:** Comparisons of mean egg collections for traps with different types and different exposure durations. (A) MOT of 3 d; (B) MOT of 7 d; (C) MOT of 10 d; (D) OT of 3 d; (E) OT of 7 d; (F) OT of 10 d. p >.

	Mean egg collection I	Mean egg collection II	t / F	*P* value
MOT vs OT with the same exposure durations	**A**: 12.38	**D**: 0.63	*t* = 8.068	<0.001
	**B**: 34.15	**E**: 2.92	*t* = 8.517	<0.001
	**C**: 50.16	**F**: 3.98	*t* = 10.021	<0.001
MOT: Different exposure durations	**A**: 12.38	**B**: 34.15	*F* = 28.911	<0.001
	**A**: 12.38	**C**: 50.16		<0.001
	**B**: 34.15	**C**: 50.16		**0.011**
OT: Different exposure durations	**D**: 0.63	**E**: 2.92	*F* = 25.405	<0.001
	**D**: 0.63	**F**: 3.98		<0.001
	**E**: 2.92	**F**: 3.98		**0.229**

**Figure 5:**
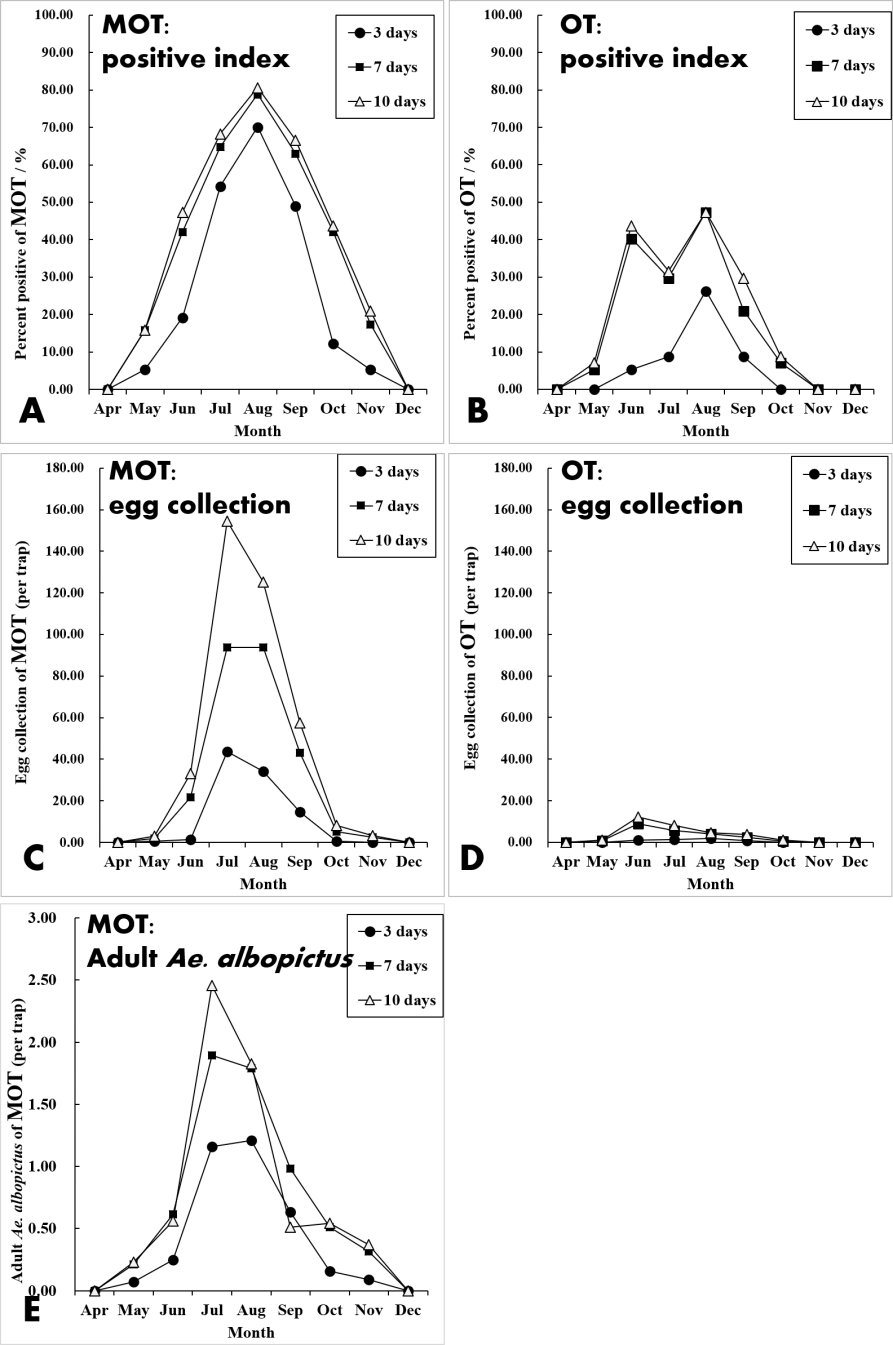
Seasonal dynamics of percent positive (%), egg collections and mosquito density of MOT and OT among three exposure durations.

Although the temporal trends of mosquito yields between MOT and OT were significantly correlated (Pearson’s correlation coefficient *r* = 0.413, *P* = 0.023), there was a difference between these traps. MOT monitoring indicated that the *Ae. albopictus* population peaked in July and August (Figs. 5A, 5C and 5E). However, the largest egg collections for OT appeared in June, and there was an obvious decline in July (Figs. 5B and 5D).

### Comparison between MOT and CLT

**Figure 6:**
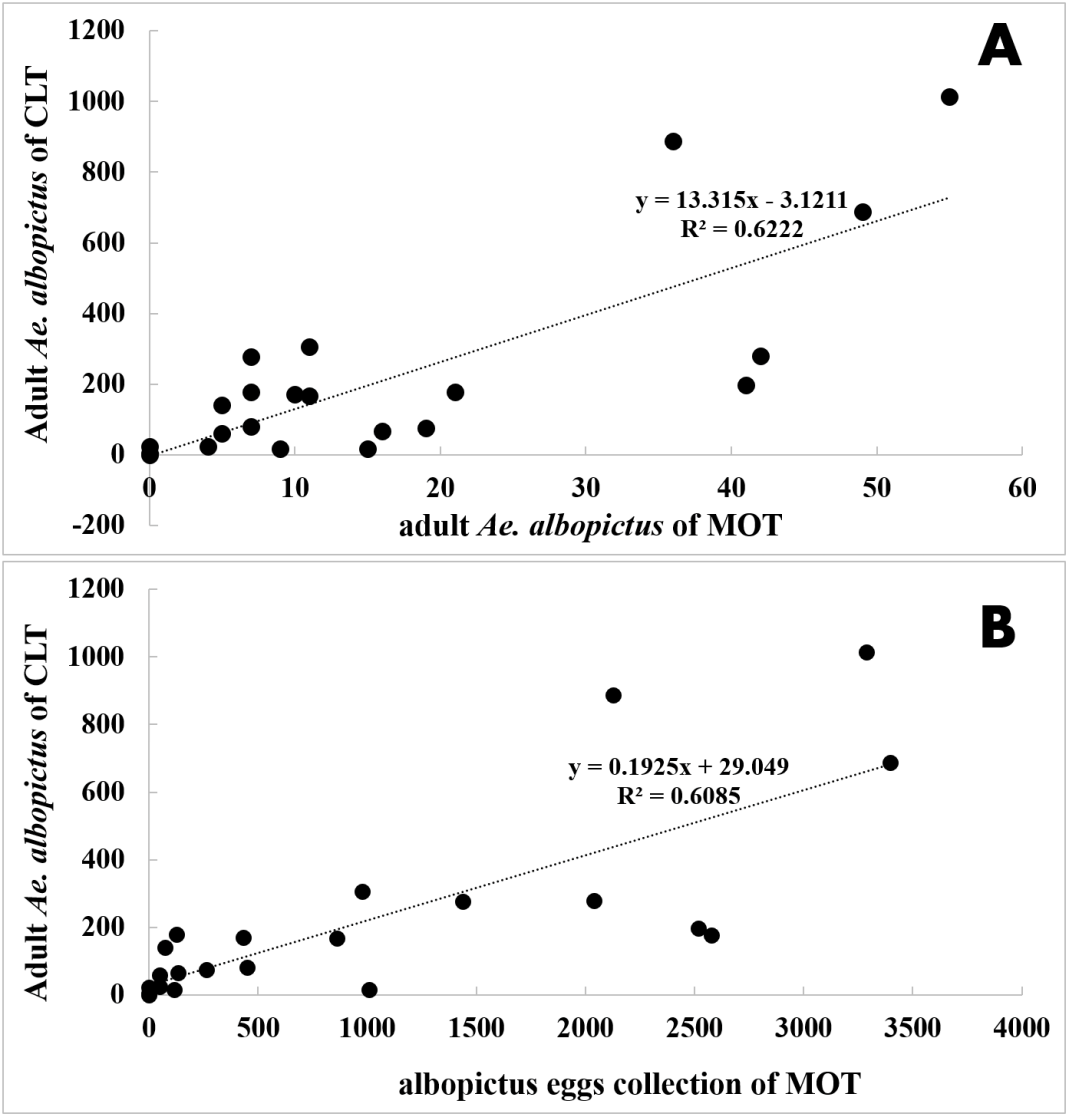
Correlation analysis between *Ae. albopictus* yields of MOT (10 d exposure duration) and CLT. (A) adult *Ae. albopictus* of CLT and adult *Ae. albopictus* of MOT; (B) adult *Ae. albopictus* of CLT and *Ae. albopictus* egg collection of MOT.

Correlation analysis showed that there were strong temporal correlations between yields of MOT and CLT at all five sampling fields and the three different MOT exposure durations (Table 3 and Fig. 6). Both traps indicated that *Ae. albopictus* populations peaked in July and started to decline in August (Table S2). CLT collected adult mosquitoes, and its efficiency in *Ae. albopictus* sampling was significantly higher than MOT (26.41 vs. 0.85 per trap, *t* =  − 3.995, *P* = 0.002).

**Table 3:** Correlation analysis between *Ae. albopictus* yields (adults and eggs) of MOT and CLT with different environmental locations.

Surveillance durations of MOT	Sites	MOT	CLT	r (pearson correlation coefficient)	*P* value
		indicators I for correlation analysis	indicators II for correlation analysis		
After 3 d	City park I	adult *Ae. albopictus*	adult *Ae. albopictus*	0.656	0.001
		*albopictus* eggs	adult *Ae. albopictus*	0.669	0.001
	City park II	adult *Ae. albopictus*	adult *Ae. albopictus*	0.596	0.003
		*albopictus* eggs	adult *Ae. albopictus*	0.715	<0.001
	3 Residential neighborhoods	adult *Ae. albopictus*	adult *Ae. albopictus*	0.492	0.02
		*albopictus* eggs	adult *Ae. albopictus*	0.801	<0.001
	Sum	adult *Ae. albopictus*	adult *Ae. albopictus*	0.582	0.005
		*albopictus* eggs	adult *Ae. albopictus*	0.610	0.003
After 7 d	City park I	adult *Ae. albopictus*	adult *Ae. albopictus*	0.699	<0.001
		*albopictus* eggs	adult *Ae. albopictus*	0.650	0.001
	City park II	adult *Ae. albopictus*	adult *Ae. albopictus*	0.583	0.004
		*albopictus* eggs	adult *Ae. albopictus*	0.439	0.041
	3 Residential neighborhoods	adult *Ae. albopictus*	adult *Ae. albopictus*	0.616	0.002
		*albopictus* eggs	adult *Ae. albopictus*	0.751	<0.001
	Sum	adult *Ae. albopictus*	adult *Ae. albopictus*	0.635	0.001
		*albopictus* eggs	adult *Ae. albopictus*	0.545	0.009
After 10 d	City park I	adult *Ae. albopictus*	adult *Ae. albopictus*	0.797	<0.001
		*albopictus* eggs	adult *Ae. albopictus*	0.850	<0.001
	City park II	adult *Ae. albopictus*	adult *Ae. albopictus*	0.645	0.001
		*albopictus* eggs	adult *Ae. albopictus*	0.610	0.003
	Residential neighborhood	adult *Ae. albopictus*	adult *Ae. albopictus*	0.672	0.001
		*albopictus* eggs	adult *Ae. albopictus*	0.836	<0.001
	Sum	adult *Ae. albopictus*	adult *Ae. albopictus*	0.789	<0.001
		*albopictus* eggs	adult *Ae. albopictus*	0.780	<0.001

**Figure 7:**
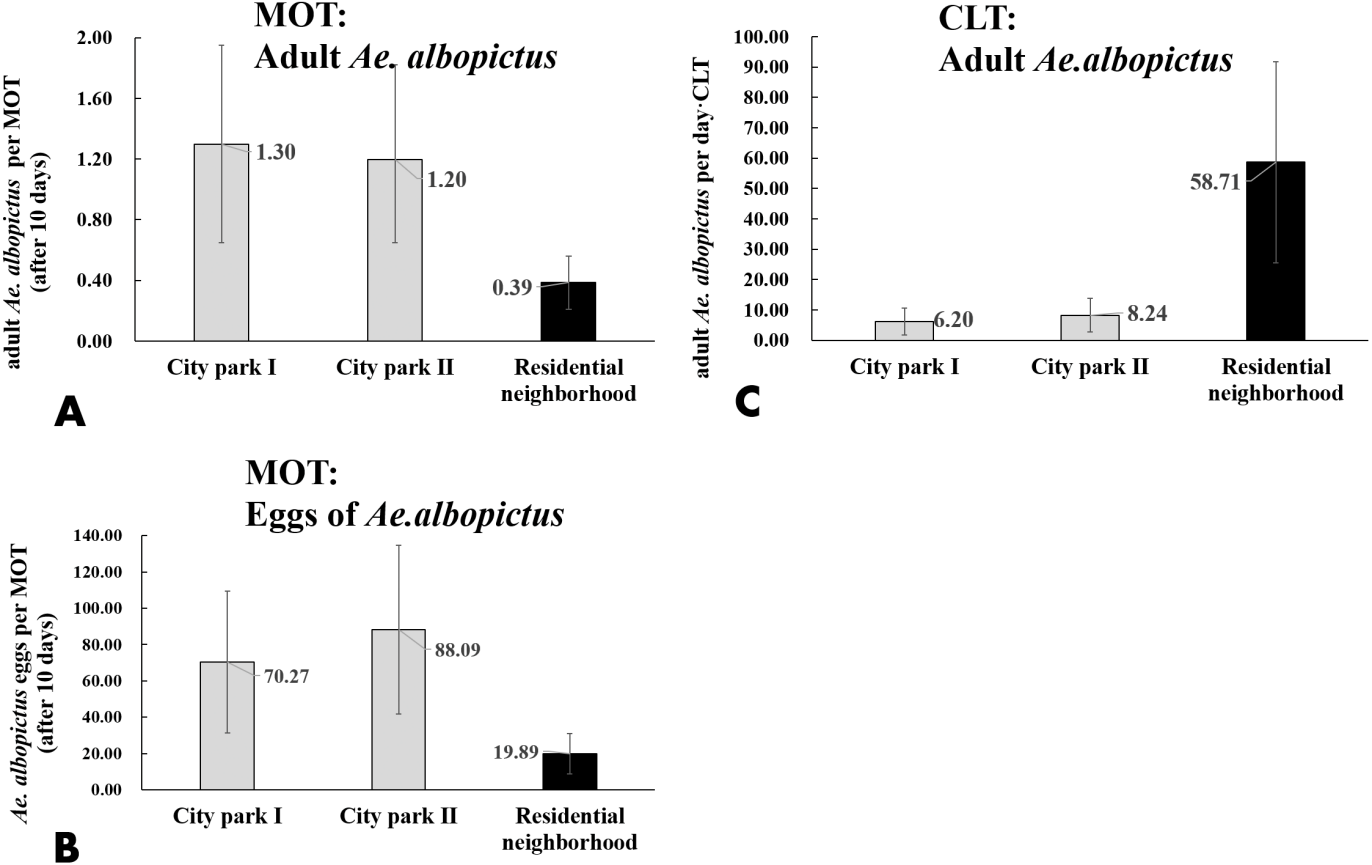
Comparison of *Ae. albopictus* yields (adults and eggs) of MOT and CLT in different environmental locations. (A) Adult *Ae. albopictus* of MOT; (B) *Ae. albopictus* eggs of MOT; (C) adult *Ae. albopictus* of CLT.

**Figure 8:**
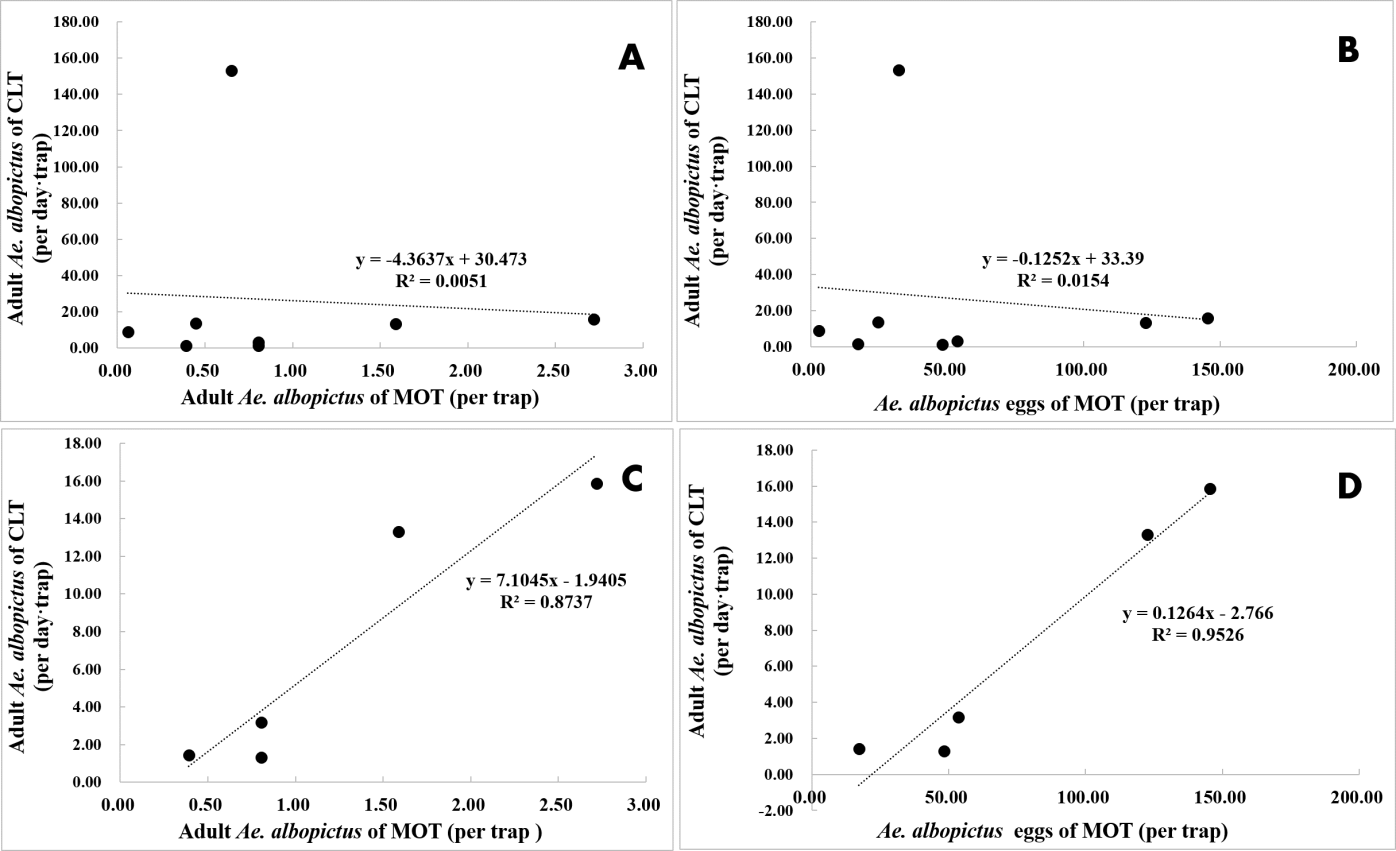
Correlation analysis between *Ae. albopictus* yields (adults and eggs) of MOT and CLT in areas with different mosquito densities. (A and B) No correlations between CLT and MOT. (C and D) Significant correlations between CLT and MOT in areas with moderate *Ae. albopictus* density.

CLT samples showed that *Ae. albopictus* were significantly more abundant in residential neighborhoods than in city parks (58.71 vs. 7.22 per day ⋅ trap, *t* =  − 3.203, *P* = 0.004). An opposite pattern was found for MOT (adults: 0.39 vs. 1.25 per trap, *t* =  − 3.203, *P* = 0.004; eggs: 19.89 vs. 79.18 per trap, *t* = 3.836, *P* < 0.001) (Table 4, Figs. 7A–7C). No significant correlations were observed among locations between the indices of CLT and MOT (correlation coefficient r = −0.124, *P* = 0.774) (Figs. 8A and 8B). However, for the 5 groups of traps located in city parks with moderate *Ae. albopictus* density (<13.77 per day ⋅ trap by CLT), a significant correlation was found between sampling yields of CLT and MOT (correlation coefficient *r* = 0.976, *P* = 0.004) (Figs. 8C and 8D).

**Table 4:** Comparison of *Ae. albopictus* yields (adults and eggs) of MOT and CLT in different environmental locations.

Sampling fields	CLT		MOT		
	Trap locations	Adult density (per day⋅ trap)	Trap locations^a^	Adult density (per trap)	Egg density (per trap)
City park I	1	1.43	1 & 2	0.39	17.17
	2	1.30	3 & 4	0.80	48.24
	3	15.87	5 & 6	2.72	145.39
City park II	4	3.17	7 & 8	0.80	53.57
	5	13.30	9 & 10	1.59	122.61
Residential neighborhoods	6	153.57	11, 12 & 13	0.65	32.14
	7	13.57	14, 15 & 16	0.45	24.68
	8	9.00	17, 18 & 19	0.06	2.86

**Notes.**

a locations of MOTs corresponding to CLTs, among the 8 locations, each CLT (total = 8 traps) was accompanied by 2 or 3 MOTs (total = 19 traps).

## Discussion

In this study, more *Ae. albopictus* eggs were collected from MOTs than OTs. We found that the minimum length of time that MOTs are exposed in the field should be at least 7 d. Significant temporal correlations were observed between sampling measurements of MOT and CLT, but there was substantial variation in the spatial distribution of *Ae. albopictus* density between MOT and CLT. MOT underestimated *Ae. albopictus* abundances in areas with high *Ae. albopictus* density compared to CLT.

Before the development of the MOT, the OT was regarded as an efficient and sensitive method for detecting the presence/absence of dengue vectors, even at low population densities (Chadee & Corbet, 1987; Evans & Bevier, 1969). MOT was designed by Lin (Lin et al., 2005), who compared MOT and OT as tools for *Ae. albopictus* monitoring. They demonstrated a positive correlation between the captures made by the two traps. However, MOT was somewhat less productive compared to OT (Lin et al., 2006a). We also observed positive correlations between the indices of MOTs and OTs, but we found that the MOT was more efficient than OTs for sampling *Ae. albopictus* eggs. Chadee (Chadee & Corbet, 1987) also reported that the number of traps receiving eggs and the number of eggs per positive trap were usually lower for OTs. Therefore, modifications were developed for trap enhancement. Hay infusion, as attractants, and hard fiberboard paddles, as an oviposition substrate, increased the eggs yields for OTs (Reiter, Amador & Colon, 1991; Ritchie, 2001). However, these modifications were not used in this study. To match the conditions of MOTs and strengthen comparability, dechlorinated tap water and filter paper oviposition substrate were used for both MOTs and OTs. Tap water and filter paper may not be optimum attractants for use in OTs. Another important reason may be attributed to the special structural design of MOTs with easy entrance access but difficult egress. Gravid *Ae. albopictus* females may hover around MOTs or OTs and hesitate before laying eggs in these artificial containers. A percentage of the females contacting OTs does not lay eggs in the trap, perhaps due to lack of suitable oviposition stimulants. A portion of females trapped by MOTs had no choice but to lay eggs inside the MOTs since they were unable to exit. This greatly increased the egg collections of the MOTs. So, under the same conditions, MOTs typically collected more eggs than OTs.

MOTs are used for mosquito surveillance in China, and the currently recommended inspection duration is 3 d. This duration was based on field tests (Lin et al., 2006a). They found that mosquito yields increased significantly with increased exposure duration, but durations longer than 3 d increased the mortality of the mosquitoes in the MOTs. We suggest that collection of live mosquitoes is less important than precise surveillance outcomes. MOTs were designed to attract oviposition-seeking females (Li et al., 2016), and a longer duration attracts more females and allows the captured gravid females enough time to lay eggs, which increases the monitoring sensitivity. The rapid increase of percent positive and egg collections during 7 d indicates that the appropriate monitoring duration for MOTs surveillance should be no less than 7 d. Moreover, a widely accepted scheme using CDC ovitraps in the field for 7 d of surveillance provides support for this suggestion (Reiter, Amador & Colon, 1991). Our tests also showed that high evaporation rates can occur during the warm summer season (July and August in Shanghai). Therefore, water needs to be replenished during the 7 monitoring days when temperatures are high.

Ovitrap data may help provide estimates of adult *Aedes* populations in some case. Mattia (Manica et al., 2017) showed that it is possible to predict the abundance of adult *Ae. albopictus* females based on egg collections. A significant positive relationship was found between the mean numbers of *Ae. albopictus* females collected by human landing catch and *Ae. albopictus* eggs collected by OTs. However, this type of positive relationship between *Ae. albopictus* eggs and adult females was not fully reproduced in the present study using MOT. As a modified ovitrap, MOT correlated with CLT in areas with moderate *Ae. albopictus* densities (<13.77 per day ⋅ trap by CLT) but underestimated *Ae. albopictus* abundances in areas with high densities (>25.56 per day ⋅ trap by CLT) compared with CLT. The latter phenomenon was consistent with the suggestion by Focks (2003) that ovitrap data cannot be reliably used to estimate the differences in adult population abundance. This result was predictable. *Ae. albopictus* has strong oviposition preference for particular sites, and this preference is governed by the combined effects of many physical and chemical stimuli that act over a range of distances (Silver, 2008). Thavara et al. (1989) demonstrated that *Ae. albopictus* prefers to lay eggs in containers holding conditioned water that has been “aged” outside for an extended period together with harboring the immature stages of this species. MOT is not a preferred oviposition site for *Ae. albopictus* compared to natural containers with conditioned water. Areas with relatively low *Ae. albopictus* density may lack natural breeding sites, and, in these areas, it is reasonable that gravid *Ae. albopictus* females would lay eggs in the artificial MOTs since there are few preferred choices. In areas with high *Ae. albopictus* density, it may be easy for them to find preferred oviposition sites, and MOTs in these areas would be less-preferred oviposition choices.

In this study, MOTs collected mostly *Ae. albopictus*, and few other species were attracted. This may have resulted because of a) the relatively limited mosquito species composition in urban Shanghai, and b) the different oviposition preferences of different mosquito species. In urban Shanghai, the predominant mosquito species are *Ae. albopictus* and *Cx. pipiens* complex. Other species like *Cx. tritaeniorhynchus* and *An. sinesis* are rare (Gao et al., 2014). Due to its design, the MOT containing water and filter paper was aimed to mainly attract gravid female mosquitoes and allow them to ovoposit (Li et al., 2016). Among the common mosquito species in downtown Shanghai, *Ae. albopictus* prefers to lay eggs in container water, while *Cx. pipiens* prefers to lay eggs in waste or polluted water. *Cx. tritaeniorhynchus* and *An. Sinesis* both prefer large water bodies like paddy fields or irrigation ditches, which are more common in rural areas. So the physical features of water and the MOT design are unsuitable for the other mosquito species of urban Shanghai.

In the field test, more data losses of OTs occurred than MOTs. The causes of data losses were primarily due to dried, tipped, and missing container and animal interference. OTs have an upper part completely exposed to the air making it possible for small animals (i.e., dogs and cats) to drink the water in the trap. During the hot summer, water inside the trap tends to evaporate rapidly. The open-design of OTs increases water evaporation compared with the semi-open design of MOT, and there is greater drying caused by water evaporation for OT in July and August compared with MOT (Figs. 4C and 4D). This may help explain the considerable variance of temporal trends of mosquito yields between MOT and OT. Flooding was a major cause for data losses for MOTs without overhead shelter. In rainy periods, excessive rain can flow into the traps and submerge the filter paper. We suggest that a drainage hole should be drilled in the middle of the MOT’s concave-bottom to reduce flooding problems. We also found spiders and geckos entering the trap and consuming mosquitoes and the eggs collected by the traps. However, snails or roaches, which also may eat or dislodge eggs, were not found in this study (Kloter, Bowman & Carroll, 1983).

MOTs have certain shortcomings. Ovitrap data cannot be reliably used to estimate the differences in adult *Ae. albopictus* abundance, and MOT data were unable to predict differences of *Ae. albopictus* population abundance among different locations. In China, percent positive of MOT < 5% or MOT positive index <5 (MOI < 5) has been used as a threshold to characterize *Ae. albopictus* density as sufficiently low as to not require control. This criterion was established based on the Breteau Index (BI: number of positive small water bodies per 100 houses inspected), which represents the positive percentage of natural breeding sites, whose threshold was also <5 (BI <5). However, the percent of positive MOTs cannot estimate or be equal to the Breteau Index, since these two indices have a competitive relationship. Even though field tests in Guangdong Province suggest that the positive index of MOTs (MOI, number of positive traps per l00 MOTs) was almost equal to Breteau Index (Lin et al., 2006c), we believe that this equivalency is probably not widely applicable. A field test in Shanghai demonstrated that significant differences exist between MOI and BI (Pan et al., 2016). Based on these arguments, we suggest that MOI cannot replace or be equal to BI. Both of these indices should be used for assessment of *Ae. albopictus* oviposition or breeding status.

The results of this study had the following shortcomings: (a) The maintenance and servicing of MOTs or OTs was labor intensive as was egg counting and species identification. CLT collection performance was also expensive. We therefore established only 8 sites for comparisons among MOTs, OTs and CLTs. However, the relatively small sample sizes provided useful initial results. More convincing evidence to support our tentative conclusion will require comprehensive, large-scale field comparisons; (b) To reduce direct competition, all traps within each site were separated by an average distance of 20 m. However, this did not completely eliminate competition. A latin-square design would have been superior, but the CLT used required a DC power supply, which limited the possible CLT locations. Despites these shortcomings, continuous sampling over 8 months provided useful information about MOTs, which can be of help in improving designs for future *Ae. albopictus* surveillance.

## Conclusions

MOT is more efficient than OT in measurements of percent positive and egg collections of *Ae. albopictus*. The minimum length of time that MOTs are deployed in the field should be at least 7 d. Strong temporal correlations were observed between sampling measurements of MOT and CLT, but there was substantial variation in the spatial distribution of *Ae. albopictus* density measured by MOT and CLT. MOT underestimated *Ae. albopictus* abundances in areas with high *Ae. albopictus* density (>25.56 per day ⋅ trap by CLT). In areas with moderate *Ae. albopictus* densities (<13.77 per day ⋅ trap by CLT), MOT data were better correlated with CLT data.

##  Supplemental Information

10.7717/peerj.8031/supp-1 File S1 Raw data of MOT, OT and CLT in different sampling date and locations

10.7717/peerj.8031/supp-2 Table S1 Percent positive (%) and egg collections of MOT and OT of different exposure durations in different locations ^a,b,c,d,e,f^ positive index corresponding to comparative parameters of Table 2. ^A,B,C,D,E,F^ index of average egg collections corresponding to comparative parameters of Table 3.

10.7717/peerj.8031/supp-3 Table S2 *Ae. albopictus* yields for MOT (after 10 d exposure) and CLT with different environmental locations **^*a*^** Mosquito yields of MOTs after 10 d exposure duration were used for comparison, which represent the maximum collections for one interval in this study.

## Additional Information and Declarations

### Field Study Permissions

The following information was supplied relating to field study approvals (i.e., approving body and any reference numbers): The field work was approved by Huangpu Center of Disease Control and Prevention, and the approval number is HPCDC-SY20170302.

### Data Availability

The following information was supplied regarding data availability: The raw measurements are available in the Supplementary Files.
