# Supplementary material for: Field evaluation of Mosq-ovitrap, Ovitrap and a CO2-light trap for Aedes albopictus sampling in Shanghai, China
Source: PeerJ. 2019 Nov 27;7:e8031. doi: 10.7717/peerj.8031 (PMC6884993; doi:10.7717/peerj.8031)
Supplement: Table S1 — a,b,c,d,e,f positive index corresponding to comparative parameters of Table 2. A,B,C,D,E,F index of average egg collections corresponding to comparative parameters of Table 3. [file peerj-07-8031-s002.docx]

| Trap  location | Percent positive / % | | | | | | |  | Egg collections / per trap | | | | | | |
| --- | --- | --- | --- | --- | --- | --- | --- | --- | --- | --- | --- | --- | --- | --- | --- |
|  | Mosq-ovitrap (MOT) | | |  | Ovitrap (OT) | | |  | Mosq-ovitrap (MOT) | | |  | Ovitrap (OT) | | |
|  | 3 d | 7 d | 10 d |  | 3 d | 7 d | 10 d |  | 3 d | 7 d | 10 d |  | 3 d | 7 d | 10 d |
| 1 | 0.00 | 4.35 | 8.70 |  | 0.00 | 0.00 | 4.35 |  | 0.00 | 0.00 | 1.39 |  | 0.00 | 0.00 | 0.87 |
| 2 | 30.43 | 47.83 | 47.83 |  | 13.04 | 30.43 | 26.09 |  | 12.91 | 23.91 | 32.96 |  | 0.57 | 4.26 | 4.78 |
| 3 | 39.13 | 60.87 | 65.22 |  | 4.35 | 17.39 | 21.74 |  | 15.00 | 39.35 | 58.43 |  | 0.22 | 1.09 | 2.39 |
| 4 | 26.09 | 43.48 | 47.83 |  | 0.00 | 17.39 | 17.39 |  | 8.30 | 26.48 | 38.04 |  | 0.00 | 1.30 | 1.52 |
| 5 | 65.22 | 69.57 | 69.57 |  | 0.00 | 21.74 | 17.39 |  | 50.26 | 109.74 | 145.57 |  | 0.00 | 2.26 | 2.39 |
| 6 | 34.78 | 69.57 | 69.57 |  | 8.70 | 17.39 | 13.04 |  | 25.17 | 78.43 | 145.22 |  | 1.22 | 3.48 | 2.39 |
| 7 | 26.09 | 43.48 | 47.83 |  | 4.35 | 13.04 | 17.39 |  | 17.57 | 59.48 | 99.57 |  | 0.39 | 0.87 | 3.78 |
| 8 | 47.83 | 69.57 | 69.57 |  | 21.74 | 30.43 | 34.78 |  | 39.83 | 113.74 | 145.65 |  | 1.83 | 6.30 | 6.78 |
| 9 | 21.74 | 30.43 | 30.43 |  | 8.70 | 13.04 | 8.70 |  | 6.96 | 10.91 | 13.26 |  | 0.61 | 1.04 | 0.61 |
| 10 | 47.83 | 56.52 | 69.57 |  | 17.39 | 39.13 | 47.83 |  | 21.57 | 76.91 | 93.87 |  | 3.00 | 7.74 | 15.91 |
| 11 | 8.70 | 21.74 | 26.09 |  | 0.00 | 17.39 | 21.74 |  | 1.09 | 4.22 | 9.61 |  | 0.00 | 2.09 | 2.30 |
| 12 | 30.43 | 39.13 | 43.48 |  | 8.70 | 39.13 | 47.83 |  | 0.57 | 3.61 | 8.22 |  | 0.22 | 4.91 | 6.70 |
| 13 | 34.78 | 47.83 | 47.83 |  | 0.00 | 13.04 | 21.74 |  | 4.48 | 13.83 | 19.22 |  | 0.00 | 1.91 | 3.96 |
| 14 | 21.74 | 56.52 | 52.17 |  | 4.35 | 17.39 | 21.74 |  | 2.17 | 9.52 | 17.26 |  | 0.87 | 4.43 | 4.65 |
| 15 | 47.83 | 60.87 | 65.22 |  | 4.35 | 39.13 | 43.48 |  | 12.30 | 43.48 | 59.96 |  | 0.87 | 7.83 | 10.52 |
| 16 | 43.48 | 52.17 | 60.87 |  | 13.04 | 30.43 | 30.43 |  | 12.52 | 27.61 | 56.22 |  | 1.00 | 4.04 | 3.39 |
| 17 | 4.35 | 13.04 | 13.04 |  | 8.70 | 13.04 | 17.39 |  | 1.74 | 2.39 | 3.26 |  | 1.17 | 1.61 | 2.48 |
| 18 | 0.00 | 0.00 | 0.00 |  | 4.35 | 4.35 | 4.35 |  | 0.00 | 0.00 | 0.00 |  | 0.09 | 0.22 | 0.22 |
| 19 | 4.35 | 17.39 | 17.39 |  | 0.00 | 0.00 | 0.00 |  | 2.83 | 5.30 | 5.30 |  | 0.00 | 0.00 | 0.00 |
| Mean | 28.15**^a^** | 42.33**^b^** | 44.85**^c^** |  | 6.41**^d^** | 19.68**^e^** | 21.97**^f^** |  | 12.38**^A^** | 34.15**^B^** | 50.16**^C^** |  | 0.63**^D^** | 2.92**^E^** | 3.98**^F^** |

^a, b, c, d, e, f^ positive index corresponding to comparative parameters of Table 2; ^A, B,C, D, E, F^ index of average egg collections corresponding to comparative parameters of Table 3.
