# Supplementary material for: Field evaluation of Mosq-ovitrap, Ovitrap and a CO2-light trap for Aedes albopictus sampling in Shanghai, China
Source: PeerJ. 2019 Nov 27;7:e8031. doi: 10.7717/peerj.8031 (PMC6884993; doi:10.7717/peerj.8031)
Supplement: Table S2 — a Mosquito yields of MOTs after 10 d exposure duration were used for comparison, which represent the maximum collections for one interval in this study. [file peerj-07-8031-s003.docx]

| ten-days | **MOT:  adult *Ae. albopictus* (after 10 d)^a^** | | |  | **MOT: *albopictus* egg-collection (after 10 d)** | | |  | **CLT: adult *Ae. albopictus*** | | |
| --- | --- | --- | --- | --- | --- | --- | --- | --- | --- | --- | --- |
|  | **City**  **park I** | **City**  **park II** | **residential neighbourhood** |  | **City**  **park I** | **City**  **park II** | **residential neighbourhood** |  | **City**  **park I** | **City**  **park II** | **residential neighbourhood** |
| Late April | 0.00 | 0.00 | 0.00 |  | 0.00 | 0.00 | 0.00 |  | 0.00 | 0.00 | 0.00 |
| Early May | 0.00 | 0.00 | 0.00 |  | 0.00 | 0.00 | 0.00 |  | 1.67 | 1.50 | 5.00 |
| Mid May | 0.17 | 0.75 | 0.56 |  | 5.00 | 11.50 | 4.44 |  | 0.00 | 2.00 | 4.33 |
| Late May | 0.00 | 0.25 | 0.33 |  | 0.00 | 0.00 | 5.56 |  | 0.00 | 4.50 | 5.00 |
| Early June | 1.00 | 0.25 | 0.00 |  | 28.67 | 35.00 | 15.11 |  | 0.67 | 11.50 | 18.67 |
| Mid June | 1.00 | 1.75 | 0.22 |  | 28.00 | 169.50 | 18.11 |  | 0.00 | 3.00 | 3.67 |
| Late June | 0.50 | 0.50 | 0.56 |  | 13.50 | 46.25 | 18.33 |  | 2.33 | 10.50 | 47.67 |
| Early July | 2.50 | 4.00 | 0.56 |  | 117.67 | 261.25 | 41.78 |  | 16.00 | 51.50 | 245.67 |
| Mid July | 4.50 | 3.25 | 1.67 |  | 225.83 | 231.25 | 111.78 |  | 32.67 | 40.50 | 278.67 |
| Late July | 5.17 | 2.75 | 0.78 |  | 314.17 | 270.50 | 47.56 |  | 36.67 | 14.00 | 183.33 |
| Early August | 2.00 | 1.50 | 0.33 |  | 182.17 | 291.25 | 35.56 |  | 6.67 | 6.00 | 48.33 |
| Mid August | 3.00 | 5.25 | 0.22 |  | 187.83 | 253.50 | 41.67 |  | 2.67 | 9.50 | 56.67 |
| Late August | 3.67 | 2.25 | 1.22 |  | 160.33 | 168.25 | 45.00 |  | 7.67 | 13.00 | 77.00 |
| Early September | 0.67 | 0.25 | 0.22 |  | 148.83 | 81.50 | 24.22 |  | 15.33 | 6.50 | 73.00 |
| Mid September | 1.00 | 0.75 | 0.22 |  | 84.50 | 47.00 | 18.33 |  | 10.00 | 5.00 | 42.33 |
| Late September | 0.50 | 1.75 | 0.11 |  | 61.67 | 126.25 | 11.33 |  | 5.33 | 1.00 | 96.33 |
| Early October | 0.50 | 0.25 | 0.33 |  | 6.17 | 6.50 | 7.11 |  | 2.00 | 4.00 | 55.00 |
| Mid October | 1.67 | 1.00 | 0.44 |  | 23.50 | 18.50 | 5.56 |  | 0.33 | 0.50 | 24.67 |
| Late October | 0.17 | 0.50 | 0.22 |  | 5.33 | 5.50 | 2.22 |  | 0.33 | 3.00 | 44.67 |
| Early November | 1.33 | 0.50 | 0.67 |  | 14.67 | 2.50 | 3.89 |  | 2.00 | 0.00 | 20.33 |
| Mide November | 0.50 | 0.00 | 0.22 |  | 8.33 | 0.00 | 0.00 |  | 0.33 | 1.00 | 19.00 |
| Late November | 0.00 | 0.00 | 0.00 |  | 0.00 | 0.00 | 0.00 |  | 0.00 | 1.00 | 1.00 |
| Early December | 0.00 | 0.00 | 0.00 |  | 0.00 | 0.00 | 0.00 |  | 0.00 | 0.00 | 0.00 |
| Mean (95% CI) | 1.30 (0.65-1.95) | 1.20 (0.58-1.82) | 0.39 (0.21-0.56) |  | 70.27 (31.16-109.38) | 88.09 (41.87-134.80) | 19.89 (8.78-31.00) |  | 6.20 (1.81-10.60) | 8.24 (2.71-13.77) | 58.71 (25.56-91.87) |

**^a^** Mosquito yields of MOTs after 10 d exposure duration were used for comparison, which represent the maximum collections for one interval in this study.
